# A genetic risk score composed of rheumatoid arthritis risk alleles, HLA-DRB1 haplotypes, and response to TNFi therapy – results from a Swedish cohort study

**DOI:** 10.1186/s13075-016-1174-z

**Published:** 2016-12-03

**Authors:** Xia Jiang, Johan Askling, Saedis Saevarsdottir, Leonid Padyukov, Lars Alfredsson, Sebastien Viatte, Thomas Frisell

**Affiliations:** 1 Unit of Clinical Epidemiology (KEP), Department of Medicine, Karolinska University Hospital, SE-171 76 Stockholm, Sweden; 2 Department of Genetic Epidemiology, Harvard T.H. Chan School of Public Health, 02115 Boston, MA USA; 3 Rheumatology Unit, Department of Medicine Solna, Karolinska Institutet, and Karolinska University Hospital, Stockholm, Sweden; 4 Cardiovascular Unit, Institute of Environmental Medicine, Karolinska Institutet, Stockholm, Sweden; 5 Arthritis Research UK Centre for Genetics and Genomics, Centre for Musculoskeletal Research, Faculty of Biology, Medicine and Health, Manchester Academic Health Science Centre, The University of Manchester, Oxford Road, Manchester, M13 9PT UK

**Keywords:** Rheumatoid arthritis risk SNPs, HLA-DRB1 haplotypes, Tumor necrosis factor inhibitor prediction, Genetic

## Abstract

**Background:**

To prevent debilitating and irreversible joint damage, rheumatoid arthritis (RA) is often treated with tumor necrosis factor inhibitor (TNFi), but many patients do not respond to this costly therapy. Few predictors for response are known, and it has been proposed that genetic factors which influence the development of RA may also influence disease severity and response to therapy. Several previous studies have attempted to confirm this but results remain inconclusive. We expand on previous studies by including more RA risk alleles, and maximize power by combining them into a genetic risk score.

**Method:**

We linked genotyped RA patients from the Epidemiological Investigation of Rheumatoid Arthritis study to the Swedish Rheumatology Quality Register, identifying patients who started a TNFi as their first biological disease-modifying anti-rheumatic drug, with a return visit within 2–8 months after treatment start (*N* = 867). We calculated risk scores from 76 established RA risk SNPs, and four HLA-DRB1 amino acid positions, and tested whether risk scores or individual genetic risk factors could predict the European League Against Rheumatism (EULAR) response.

**Results:**

We found no association between any of the risk scores or HLA-DRB1 haplotypes and EULAR response, neither overall nor stratified by anti-citrullinated protein/peptide antibody (ACPA) status. When evaluating each of the 76 SNPs, we found that the number of SNPs presenting significant associations was not higher than expected by chance (5/76 SNPs had *p* < 0.05 in ACPA-positive RA, 4/76 in ACPA-negative RA).

**Conclusion:**

Overall, known RA risk SNPs do not predict response to TNFi, either individually or when combined into a risk score. This does not support the hypothesis that genes influencing RA onset would also influence its prognosis and treatment response.

**Electronic supplementary material:**

The online version of this article (doi:10.1186/s13075-016-1174-z) contains supplementary material, which is available to authorized users.

## Background

Tumor necrosis factor inhibitor (TNFi) therapy represents an important breakthrough in the treatment of the chronic inflammatory joint disease rheumatoid arthritis (RA). Although the therapeutic utility of TNFi is well documented, the drugs are costly and patients display substantial heterogeneity in treatment response: approximately 30% of patients discontinue therapy in the first year, a decision often made due to lack of effect or adverse events [1].

In order to personalize treatment, substantial efforts have been made to identify factors predicting response to TNFi. Environmental or clinical factors such as smoking, gender, age, baseline disability, or presence of autoantibodies account for only a small proportion of the variability in patient response [2, 3]. Unfortunately, genome-wide association studies (GWASs) have so far not revealed any clear evidence of predictive genetic markers, except for a large collection of nominally significant markers [4–8], or a few markers which, despite having approached acceptable levels of significance, are too weak to inform any clinical decisions (such as rs6427528 on CD84 with *p* = 8 × 10^–8^ in the etanercept subsets of patients) [9].

Before the advent of GWASs, studies of treatment response commonly focused on individual candidate markers. This remains a viable strategy for conserving power by reducing the number of simultaneous tests, if good candidates can be found. Candidate genes in this context have mainly come in two types: those involved in TNF metabolism or mechanistic pathways, and those associated with the onset of RA. Several investigations with limited samples (less than 130 subjects) have been conducted for the –308G > A polymorphism in the *TNF* gene [10–18], and found that the GG genotype is associated with a better response to TNFi treatment [11, 16–18]. This finding, however, was neither supported by a following study with a larger sample [19] nor replicated in subsequent genome-wide interrogations [4–7, 9].

Several studies have focused instead on individual genes known to be associated with risk for RA, including *HLA-DRB1*, *PTPN22*, *IL*, and *FCGR*, but no statistically significant associations were found [2, 19–22], except for one study finding two copies of *HLA-DRB1* shared epitope (SE) significantly associated with improved ACR50 response [23] and a recent study investigating amino acid positions, rather than the SNPs per se, finding that valine in amino acid position 11 in *HLA-DRB1* (which is outside the well-described SE positions) was associated with radiological progression and response to TNFi [24].

GWASs have provided researchers with an expanded list of RA risk genes. In a study of the then 31 identified RA risk loci, only one SNP (rs10919563) on *PTPRC/CD45* was associated with European League Against Rheumatism (EULAR) response and DAS28 changes among TNFi starters, with reasonable replications and acceptable sample sizes (ca. 1200 in each study) [25, 26]. With some possible exceptions, including the HLA region, this observation may suggest that most alleles contributing to the development of RA do not influence treatment response when the disease has been established, and would be in line with the finding that a family history of RA (a proxy of genetic liability to develop RA) does not predict RA TNFi treatment response [27]. It could also be argued, however, that previous studies were underpowered, and only focused on a subset of the now identified genetic markers for RA.

We hypothesized that many alleles associated with the development of RA are also important in predicting TNFi response, albeit with small individual effects, and that the previous failure to show this was due to lack of power. To extend previous research, we included more RA risk alleles and maximized power by combining these multiple polymorphisms into a single parameter—a genetic risk score. We analyzed these genetic markers (all currently known RA risk SNPs tagged by the Immunochip platform, and genome-wide SNPs) and several HLA-DRB1 amino acids (positions 11, 13, 71, and 74, and haplotypes defined by these positions), grouped into scores as well as individually, to evaluate how well they predict response to TNFi therapy among patients with RA who used TNFi as their first biological disease-modifying anti-rheumatic drug (bDMARD).

## Methods

We performed a cohort study in prospectively recorded data by linking all participants in the Epidemiological Investigation of Rheumatoid Arthritis (EIRA) incident case–control study, who had been genotyped with the Immunochip array, to the Swedish Rheumatology Quality Register (SRQ), identifying patients starting TNFi therapy as their first bDMARD and their response to this treatment.

### Epidemiological Investigation of Rheumatoid Arthritis

EIRA is a population-based case–control study initiated in 1996. Cases were recruited from all rheumatology providers within defined areas in Sweden, within 1 year of symptom onset and initial visit to a rheumatologist. At baseline, participants completed a self-administrated questionnaire and provided blood samples for serologic (anti-citrullinated protein/peptide antibodies (ACPA)) and genetic examinations. A total of 5043 EIRA subjects (all were recruited until 2009) were available on Immunochip genotypes; after quality control, 4830 were eligible for amino acid imputation. Imputation on amino acid positions 11, 13, 71, and 74 as well as the haplotypes based on the four positions was finally successfully performed for 4726 participants (2785 patients and 1941 controls). We subsequently linked the EIRA patients with the SRQ to further identify the target patient population of the current study. All participants consented to be involved in the study.

### Swedish Rheumatology Quality Register

The SRQ is a profession-driven, web-based national quality register engaging both patients and rheumatologists. This clinical register records longitudinal data entered by the patient and the treating rheumatologist at each visit. The nationwide coverage is good for patients with newly diagnosed RA (about 80%), and is excellent for RA patients treated with bDMARDs (about 90%). Of the aforementioned 2785 EIRA patients with genetic information, 2576 were registered in the SRQ (92%); 895 were registered as starting any of the five TNFi agents (etanercept, infliximab, adalimumab, certolizumab pegol, and golimumab) as their first bDMARD. Finally, a total of 867 subjects (653 ACPA-positive patients and 165 ACPA-negative patients) who had a valid visit registered in the SRQ within 7 days of starting therapy were included in the current study.

### Exposures

We collected genetic information on the target patient population in four forms. First, we identified RA patients with the classical *HLA-DRB1* SE, genotyped as described previously [28]. Briefly, carriers of any of the HLA-DRB1*01 (but not HLA-DRB1*0103), HLA-DRB1*04 (HLA-DRB1*0401, HLA-DRB1*0404, HLA-DRB1*0408), and HLA-DRB1*10 alleles were defined as exposed; carriers of none of these alleles were defined as unexposed.

Second, we collected data on 76 RA risk SNPs as described previously (Additional file 1: Table S1) [29]. Briefly, based on the results of a meta-analysis of Immunochip data for 11,475 cases and 15,870 controls of European ancestry (which identified 46 RA risk SNPs), as well as a trans-ethnic meta-GWAS of 29,880 cases and 73,758 controls of European and Asian ancestry (which provided an additional 49 RA risk SNPs/loci), we managed to obtain data using our Immunochip material for all 46 SNPs from the first study, and an additional 14 SNPs from the second study; of the remaining 35 SNPs in the second study, proxy SNPs with *r* ^2^ > 0.6 were identified on 16 of them, resulting in a total of 76 SNPs. We constructed a weighted genetic risk score (GRS) by summing the alleles for each individual, weighted by the logarithm of the published odds ratio (OR) in the corresponding meta-GWAS, according to the following equation:

$$ {\mathrm{GRS}}_{76\mathrm{SNPs}}={\displaystyle \sum_{i=1}^{76}}\left({\mathrm{lnOR}}_{\mathrm{SNPi}}\right)\times {\mathrm{Copy}}_{\mathrm{SNPi}} $$

Third, in addition to the 76 RA risk SNPs, we further made use of the whole Immunochip and calculated a series of GRSs based on genome-wide association with being an RA patient, estimated in the full EIRA material. Those GRSs included SNPs with decreasing magnitudes of effects—from the most stringent RA associations (genome-wide significance), to the moderate associations (e.g., *p* < 0.0005, *p* < 0.005, *p* < 0.05), to the least associated (all Immunochip markers)—and were weighted by the logarithm of ORs derived from the corresponding association analysis.

Finally, the four amino acid positions 11 (six polymorphisms), 13 (six polymorphisms), 71 (four polymorphisms), and 74 (five polymorphisms) at *HLA-DRB1*, as well as the haplotypes defined by those four positions, were imputed as described previously (Additional file 2: Table S2) [30]. Briefly, the dosages of HLA amino acids were imputed from the Immunochip data with a publicly released reference panel generate by the Type 1 Diabetes Genetics Consortium using SNP2HLA software [31]. Long-range haplotypes across the MHC were obtained, which were used to extract the corresponding haplotypes and amino acid residues. We calculated the GRS for amino acid positions by summing up the dosages of residues for each individual (each person could have two residues at each of the four positions), weighted by the logarithm of its reported association to develop RA (measured with the OR); similarly, we calculated the GRS for haplotypes by weighting each haplotype based on its reported OR [32], according to the following equations:

$$ \begin{array}{l}{\mathrm{GRS}}_{\mathrm{AA}\ \mathrm{position}\left(\mathrm{i}\right)}={\displaystyle \sum_{n=1}^{no.\kern0.5em  residues\  in\  position(i)}}\left({\mathrm{lnOR}}_{\mathrm{residue}\ \mathrm{in}\ \mathrm{position}\left(\mathrm{i}\right)}\right)\times {\mathrm{Dosage}}_{\mathrm{residue}\ \mathrm{in}\ \mathrm{position}\left(\mathrm{i}\right)}\\ {}{\mathrm{GRS}}_{\mathrm{haplotype}\mathrm{s}}={\displaystyle \sum_{i=1}^2}\left({\mathrm{lnOR}}_{\mathrm{haplotype}\left(\mathrm{i}\right)}\right)\times {\mathrm{Copy}}_{\mathrm{haplotype}\left(\mathrm{i}\right)}\end{array} $$

### Outcomes

We collected the clinical characteristics for all of the eligible RA patients at treatment start and an evaluation visit, defined as a visit within 2–8 months after starting therapy, closest to 5 months if patients had multiple follow-up visits with valid DAS28 data. Disease activity was measured with the DAS28, visual analog scale (VAS) pain, Health Assessment Questionnaire (HAQ), erythrocyte sedimentation rate (ESR), C-reactive protein (CRP), swollen joint count (SJC), tender joint count (TJC), and VAS global health. We adopted two main outcomes based on these measurements: EULAR response, categorized as good/moderate vs none; and changes in the disease activity measures, calculated by subtracting the baseline values from the values at the evaluation visit, used in a continuous fashion (e.g., ΔDAS28, ΔCRP, etc.). In addition, to test the validity of GRS, we also estimated how the distribution of the GRS differed by RA status (RA vs controls).

### Analysis

We analyzed the GRS of SNPs, amino acids, and haplotypes, both as linear covariates (continuous) and categorical covariates (divided into four categories according to quartiles among controls). We also directly assessed individual prediction from each SNP, amino acid, and haplotype (i.e., in its original format: 0/1/2 copies). We assessed the association between achieving good/moderate vs no EULAR response and GRS through logistic regressions. We evaluated the association between the changes of disease activity and GRS using R-square (*R* ^2^) values from linear regressions, given that delta values are virtually normally distributed even without transformation. Two post-hoc sensitivity analyses were made, where the main analysis (categorical GRS as predictor of EULAR response) was made first excluding etanercept to assess whether the prediction was different when focusing on monoclonal antibody therapies, and second restricting the time window for the evaluation visit to 2–5 months (60–150 days) to assess whether the broad time window masked a specific association to response at that time point. Finally, to illustrate the validity of the RA GRS in this dataset, its association with RA risk (comparing the RA cases with controls) was estimated by logistic regressions. All analyses were performed by adjusting for age, gender, and five principal components (PCs, to correct for population stratification [33]) as well as stratified by ACPA status.

## Results

### Clinical presentations of RA patients

Among the 867 RA patients who started TNFi as their first bDMARD, 75% were ACPA-positive and 74% were females. At the start of TNFi treatment, we identified comparable clinical presentations between the two subsets, except for a lower ESR (24.2 vs 29.0, *p* = 0.0068) and a slightly higher TJC (8.9 vs 7.5, *p* = 0.0074) among ACPA-negative patients. At follow-up, the improvement in ACPA-positive patients and ACPA-negative patients was similar for all outcome measures (Table 1).

**Table 1:** Clinical characteristics at start of TNFi treatment and changes of outcome measures at follow-up visit (2–8 months) among RA patients overall, and stratified by ACPA status

Characteristic	Overall RA	ACPA-positive RA	ACPA-negative RA
Number of observations	867	653	165
Age, mean	51.2	51.3	51.5
Female (%)	74.1	72.1	79.2
Baseline values, mean			
DAS28	5.0	5.0	5.0
CRP	21.0	21.9	19.1
ESR	27.9	29	24.2
SJC	7.8	7.8	8.1
TJC	7.8	7.5	8.9
VAS global health	54.1	53.8	54.5
VAS pain	53.5	53.4	53.5
HAQ	1.0	1.0	1.0
Changes from baseline to follow-up visit, mean			
ΔDAS28	–1.5	–1.5	–1.6
ΔCRP	–10.2	–10.4	–9.3
ΔESR	–9.2	–9.5	–8.2
ΔSJC	–4.9	–4.8	–5.3
ΔTJC	–4.3	–4.1	–4.9
ΔVAS global health	–21.1	–21.7	–19.2
ΔVAS pain	–21.6	–22.3	–19.7
ΔHAQ	–0.3	–0.3	–0.3
EULAR response (%)			
No	25.2	25.6	23.6
Moderate	31.4	30.6	33.1
Good	43.4	43.8	43.3

*ACPA* anti-citrullinated protein/peptide antibodies, *DAS28* disease activity score 28, *CRP* C-reactive protein, *ESR* erythrocyte sedimentation rate, *RA* rheumatoid arthritis, *SJC* swollen joint count, *TJC* tender joint count, *TNFi* tumor necrosis factor inhibitor, *VAS* visual analog scale, *HAQ* Health Assessment Questionnaire, *EULAR* European League Against Rheumatism

### GRS and RA risk

We found that not all of the GRSs followed normal distributions—the risk score on position 11 was slightly skewed, and on position 13 was collapsed into three levels (Additional file 3: Table S3)—indicating a need to analyze the amino acids individually. We examined the associations between GRS and RA risk. As expected, both the linear and categorical GRS only consistently increased ACPA-positive RA risk but not ACPA-negative RA risk (Table 2 and Additional file 4: Table S4).

**Table 2:** Associations between genetic risk scores and overall RA risk, as well as ACPA-positive and ACPA-negative RA risk

GRS	Overall RA	ACPA-positive RA	ACPA-negative RA
GRS_76SNP_			
Linear	2.03 (1.85–2.23)	2.44 (2.19–2.72)	1.21 (1.03–1.44)
Q1	Reference	Reference	Reference
Q2 vs Q1	1.40 (1.02–1.93)	1.57 (1.04–2.38)	1.25 (0.77–2.05)
Q3 vs Q1	2.39 (1.78–3.22)	3.43 (2.36–5.00)	1.33 (0.81–2.16)
Q4 vs Q1	4.99 (3.77–6.61)	8.01 (5.60–11.45)	1.49 (0.92–2.42)
GRS_Haplotype_			
Linear	2.13 (1.94–2.34)	2.57 (2.30–2.87)	1.19 (1.00–1.42)
Q1	Reference	Reference	Reference
Q2 vs Q1	1.68 (1.21–2.34)	2.70 (1.69–4.33)	1.19 (0.74–1.91)
Q3 vs Q1	3.64 (2.72–4.88)	8.11 (5.34–12.31)	1.06 (0.65–1.71)
Q4 vs Q1	7.08 (5.24–9.57)	16.11 (10.51–24.69)	1.47 (0.89–2.41)

Data presented as odds ratio (95% confidence interval)

*ACPA* anti-citrullinated protein/peptide antibodies, *GRS* genetic risk score, *RA* rheumatoid arthritis, *Q*, quartile

### GRS and TNFi treatment response

In contrast, we found no association between GRS (either from the 76 SNPs, the four amino acids, or the haplotypes) and good/moderate EULAR response in RA overall or in ACPA-positive RA. Neither did the primary RA genetic risk factor, SE, predict EULAR response (Table 3). Post-hoc sensitivity analysis showed virtually identical results when excluding etanercept (Additional file 5: Table S5). Post-hoc sensitivity analysis restricting the evaluation time window to months 2–5 did not find an association with the SNP-based or haplotype-based GRS, but did find a borderline significant reduced EULAR response associated with SE alleles (any SE allele OR = 0.69 (0.49–0.97), *p* = 0.0347, full table in Additional file 6: Table S6). The second quartile of GRS at amino acid position 13 appeared to be significantly associated with improved EULAR response in ACPA-negative RA, possibly due to low numbers in this stratum (Additional file 7: Table S7). We then evaluated each of the 76 SNPs, each of the residues at the amino acid positions, and each of the haplotypes associated with EULAR response separately. The number of SNPs presenting significant associations was not higher than expected by chance (5/76 SNPs had *p* < 0.05 in ACPA-positive RA, 4/76 in ACPA-negative RA) (Additional file 8: Table S8 and Additional file 9: Figure S1). Similarly, despite the borderline significance displayed by some residues in ACPA-positive RA (glycine at position 11, serine and tyrosine at position 13) and in ACPA-negative RA (proline at position 11, arginine at position 13), none survived multiple testing corrections (Additional file 10: Table S9, 21 tests performed). In addition, none of the haplotypes, either based on four amino acids (11/13/71/74) or on three amino acids (11/71/74, 11 and 13 are in high LD), were significantly associated with good/moderate EULAR response in RA overall or ACPA-positive RA, except for a few suggestive significances in ACPA-negative RA (Table 4). We additionally performed the haplotype analyses among patients with high disease activity (baseline DAS28 > 5.1) but did not identify any significant associations (Additional file 11: Table S10). Furthermore, the sensitive analysis comparing patients with good EULAR response with those with no response (good vs no) did not reveal further evidence of associations (Additional file 12: Table S11).

**Table 3:** Associations between genetic risk scores and TNFi treatment response (achieving good/moderate EULAR response vs no) in overall RA, as well as in ACPA-positive RA and ACPA-negative RA

GRS	Overall RA	ACPA-positive RA	ACPA-negative RA
GRS_76SNPs_			
Linear	0.96 (0.81–1.14)	1.00 (0.82–1.22)	0.89 (0.56–1.42)
Q1	Reference	Reference	Reference
Q2 vs Q1	0.68 (0.31–1.50)	1.00 (0.36–2.81)	0.66 (0.17–2.54)
Q3 vs Q1	0.77 (0.37–1.60)	1.07 (0.42–2.71)	0.60 (0.15–2.36)
Q4 vs Q1	0.79 (0.39–1.57)	1.06 (0.44–2.55)	0.95 (0.23–3.93)
GRS_Haplotype_			
Linear	0.92 (0.77–1.12)	0.96 (0.76–1.20)	1.30 (0.80–2.12)
Q1	Reference	Reference	Reference
Q2 vs Q1	1.15 (0.51–2.61)	0.76 (0.22–2.65)	2.35 (0.67–8.20)
Q3 vs Q1	1.05 (0.52–2.12)	0.89 (0.30–2.65)	2.19 (0.58–8.30)
Q4 vs Q1	0.99 (0.48–2.02)	0.84 (0.27–2.54)	3.53 (0.88–14.22)
Any SE	0.79 (0.59–1.06)	0.84 (0.59–1.20)	1.07 (0.53–2.17)

Data presented as odds ratio (95% confidence interval)

*ACPA* anti-citrullinated protein/peptide antibodies, *GRS* genetic risk score, *RA* rheumatoid arthritis, *Q* quartile, *SE* shard epitope, *TNFi* tumor necrosis factor inhibitor, *EULAR* European League Against Rheumatism

**Table 4:** Associations between individual HLA-DRB1 haplotype and achieving good/moderate EULAR response, in overall RA, as well as in ACPA-positive RA and ACPA-negative RA

Haplotype	Prevalence (%)	Overall RA	ACPA-positive RA	ACPA-negative RA
PRAA	10.2	Reference	Reference	Reference
DFRE	1.7	1.60 (0.51–5.03)	1.60 (0.48–5.34)	NA
GYRQ	4.8	0.59 (0.29–1.19)	0.64 (0.28–1.47)	0.18 (0.03–1.28)
LFEA	0.4	0.23 (0.04–1.55)	NA	NA
LFRA	14.9	0.89 (0.51–1.56)	0.93 (0.49–1.79)	0.33 (0.06–1.88)
PRRA	0.8	0.89 (0.20–3.94)	2.15 (0.23–20.28)	NA
SGRA	1.7	0.72 (0.26–1.96)	0.81 (0.27–2.47)	0.28 (0.01–5.30)
SGRL	2.9	1.00 (0.41–2.43)	1.23 (0.37–4.10)	**0.14 (0.02–0.95)**
SSEA	5.4	0.92 (0.45–1.86)	1.31 (0.53–3.28)	**0.12 (0.02–0.73)**
SSKA	0.7	2.49 (0.29–21.30)	NA	0.12 (0.01–2.59)
SSKR	9.3	0.99 (0.47–2.10)	1.23 (0.51–2.99)	**0.04 (0.00–0.39)**
SSRA	3.6	1.98 (0.78–5.00)	2.43 (0.81–7.32)	0.55 (0.06–5.03)
SSRE	1.1	1.59 (0.32–7.82)	2.58 (0.30–22.14)	0.10 (0.00–2.41)
VFRA	2.1	0.52 (0.21–1.24)	0.60 (0.22–1.63)	0.17 (0.01–2.84)
VHEA	0.3	1.14 (0.11–12.24)	1.58 (0.14–17.89)	NA
VHKA	27.0	0.78 (0.46–1.32)	1.08 (0.59–1.97)	**0.16 (0.03–0.93)**
VHRA	11.9	1.10 (0.62–1.97)	1.20 (0.62–2.33)	0.93 (0.11–8.00)
VHRE	1.3	1.03 (0.31–3.43)	2.11 (0.43–10.35)	**0.03 (0.00–0.55)**
PAA	10.2	Reference	Reference	Reference
DRE	1.7	1.59 (0.51–5.01)	1.56 (0.47–5.20)	NA
GRQ	4.8	0.59 (0.29–1.19)	0.63 (0.27–1.45)	0.19 (0.03–1.33)
LEA	0.4	0.23 (0.03–1.50)	NA	NA
LRA	14.9	0.89 (0.51–1.55)	0.93 (0.49–1.77)	0.34 (0.06–1.92)
PRA	0.8	0.90 (0.20–3.98)	2.11 (0.22–19.84)	NA
SEA	5.4	0.95 (0.47–1.91)	1.36 (0.55–3.39)	**0.13 (0.02–0.75)**
SKA	0.7	2.62 (0.31–22.35)	NA	0.13 (0.01–2.73)
SKR	9.3	1.01 (0.48–2.13)	1.26 (0.52–3.05)	**0.05 (0.00–0.43)**
SRA	5.3	1.34 (0.64–2.79)	1.51 (0.65–3.52)	0.48 (0.06–3.63)
SRE	1.1	1.52 (0.31–7.42)	2.47 (0.29–21.15)	0.10 (0.00–2.51)
SRL	2.9	0.94 (0.39–2.27)	1.20 (0.36–3.98)	**0.13 (0.02–0.92)**
VEA	0.3	1.15 (0.11–12.39)	1.56 (0.14–17.60)	NA
VKA	27.0	0.77 (0.46–1.30)	1.06 (0.58–1.94)	**0.15 (0.03–0.91)**
VRA	14.0	0.95 (0.55–1.64)	1.04 (0.55–1.94)	0.64 (0.09–4.31)
VRE	1.3	1.03 (0.31–3.44)	2.08 (0.42–10.27)	**0.04 (0.00–0.66)**

*ACPA* anti-citrullinated protein/peptide antibodies, *RA* rheumatoid arthritis, *EULAR* European League Against Rheumatism

We further evaluated the performance of GRS and SE in the changes of disease activity measures. As shown in Table 5 and Additional file 13: Table S12, the only consistent association with nominal significance lay in ΔHAQ in ACPA-positive RA, where the GRS together with SE explained approximately 5% of variance (range: 3.4–5.4%), although this significance would not withstand correction for the number of tests (eight tests performed per amino acid position). Moreover, amino acid positions 11 and 13 appeared to explain a moderate proportion of variance for ΔESR (ca. 11%) in ACPA-negative RA, but this was not significant after corrections for multiple tests (eight tests performed per amino acid position). In addition, we found no evidence supporting significant associations (that withstood multiple corrections) between GRS (composed of 76 SNPs) and any of the baseline clinical characteristics (Additional file 14: Table S13).

**Table 5:** Variance in disease activity changes by genetic risk score, SE and both, in overall RA and in ACPA-positive RA and ACPA-negative RA

Changes from baseline	Overall RA		ACPA-positive RA		ACPA-negative RA	
	*R* ^2^	*p*	*R* ^2^	*p*	*R* ^2^	*p*
GRS_76SNPs_						
ΔDAS28	0.016747	0.14	0.016816	0.32	0.031191	0.77
ΔCRP	0.008274	0.63	0.007312	0.84	0.098850	0.08
ΔESR	0.005006	0.88	0.003705	0.98	0.063739	0.32
ΔSJC	0.003122	0.96	0.005690	0.91	0.052132	0.44
ΔTJC	0.009859	0.49	0.009595	0.71	0.032586	0.75
ΔVAS global	0.007589	0.67	0.007651	0.82	0.024461	0.87
ΔVAS pain	0.012959	0.30	0.013727	0.48	0.027700	0.82
ΔHAQ	0.019911	0.10	**0.034497**	*0.03*	0.019770	0.93
GRS_76SNPs_ + GRS_AAs_ + SE						
ΔDAS28	0.025706	0.50	0.034035	0.52	0.107155	0.70
ΔCRP	0.016461	0.89	0.014948	0.98	0.159459	0.28
ΔESR	0.017487	0.87	0.016454	0.98	0.104736	0.74
ΔSJC	0.007558	1.00	0.017127	0.96	0.136713	0.42
ΔTJC	0.020931	0.71	0.020560	0.91	0.095691	0.79
ΔVAS global	0.019875	0.76	0.021503	0.89	0.131984	0.47
ΔVAS pain	0.025917	0.50	0.030989	0.63	0.140222	0.39
ΔHAQ	0.029739	0.41	0.054253	0.12	0.115604	0.68

*AA* amino acid, *ACPA* anti-citrullinated protein/peptide antibodies, *GRS* genetic risk score, *RA* rheumatoid arthritis, *SE* shared epitope, *DAS28* disease activity score 28, *CRP* C-reactive protein, *ESR* erythrocyte sedimentation rate, *SJC* swollen joint count, *TJC* tender joint count, *VAS* visual analog scale, *HAQ* Health Assessment Questionnaire

### Genome-wide polygenic risk score

**Fig. 1:**
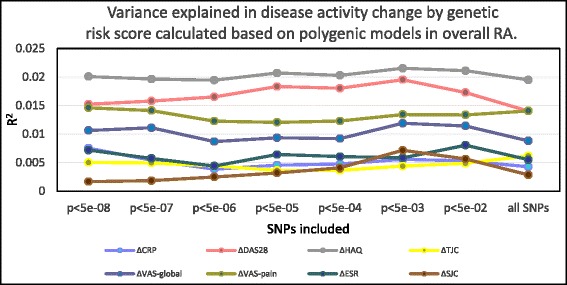
Variance in disease activity change explained by GRS calculated based on polygenic models in overall RA. *x* axis, *p*-value thresholds of SNPs included to calculate a genetic risk score. *y* axis, *R* ^2^ values calculated from the polygenic model. Eight different clinical measures are examined: ∆CRP, ∆DAS28, ∆HAQ, ∆TJC, ∆VAS-global, ∆VAS-pain, ∆ESR, ∆SJC. *Dots* map the *R* ^2^ value calculated from the polygenic model, modeling clinical measures as outcomes and the corresponding genetic risk score as the independent variable. *DAS28* disease activity score 28, *CRP* C-reactive protein, *ESR* erythrocyte sedimentation rate, *SJC* swollen joint count, *TJC* tender joint count, *VAS* visual analog scale, *HAQ* Health Assessment Questionnaire

The null findings already presented included markers that were RA risk SNPs achieving genome-wide significance in previous studies, and weaker RA risk alleles remain to be identified, which may have an effect on treatment response. Several lines of evidence have demonstrated that currently known RA susceptibility loci only account for a small proportion of ACPA-positive RA heritability, even less in ACPA-negative RA; and that by including additional common SNPs, the proportion of RA genetic liability could be explained to a larger extent [34]. We hypothesized that the *R* ^2^ estimates would be improved by incorporating extra SNPs. We therefore calculated several GRSs including SNPs with different magnitudes of association to RA, demonstrated their predictive capacity with regards to RA risk, and then assessed the association with response to TNFi using these scores; the results are plotted in Fig. 1. We found no visible trends that *R* ^2^ would increase as numbers of incorporated markers increased.

## Discussion

By linking clinical data from the Swedish Rheumatology Register to genetic data from the EIRA study, we evaluated whether known RA susceptibility genes, HLA-DRB1 amino acids, and haplotypes, either individually or integrated into a risk score, predicted treatment response to TNFi therapy. Although all were strong predictors of RA, a high genetic risk score was not associated with good EULAR response in either overall RA or stratified by ACPA status.

Our results are in line with, but also extend, the previous GWAS and candidate gene approach findings, and indicate that although treatment response in RA has been reported to be somewhat heritable [35], the genetic variants that influence disease onset do not necessarily influence TNFi treatment response to the same extent. This is also supported by a recent study showing that family history of RA did not predict RA TNFi treatment response [27], although this on its own could have been due to the information on genetic risk contained in a family history being too slight among patients who are all at high genetic risk, as evident from them having already developed the disease.

The SNP that was found to be significantly associated with both RA risk and TNFi treatment response (PTPRC/CD45 rs10919563 at chromosome 1) in a previous larger sample [25] could unfortunately not be replicated in our study because of lack of proxies (the closest possible SNP to the PTPRC region available in our material is 100 kbp distant). Even though weak yet nominally significant associations were indeed identified on a handful of individual genetic markers in the current study, they were neither strong enough to withstand correction for multiple testing nor close enough to be clinically informative. Unfortunately, this has also been the case in previous studies. For example, the first TNFi treatment response GWAS performed in 89 RA patients by Liu et al. [5] provided a reference list of 16 candidate SNPs with suggestive significance; none was replicated in a subsequent separate study with slightly larger samples (*n* = 151) [36]. Plant et al. [6] performed a multistage GWAS in 1285 RA patients and found seven genetic loci that might influence treatment response; none survived the two additional replication attempts [4, 7]. Similarly, the majority of the markers identified by Krintel et al. [4] in 196 Danish RA patients did not withstand replication in another 315 Spanish subjects [37]. However, one SNP (rs3794271) at *PDE3A-SLCO1C1* reached genome-wide significance in the meta-analysis combining the Danish and Spanish cohorts. Further investigations are needed for this pharmacogenetics biomarker of interest. GWASs performed after Liu et al. attempted to both identify new predictors and replicate previous findings, neither of which succeeded. This may be due to the small sample size for GWASs, where often 2–5 million SNPs were analyzed with an average sample size less than 1000. We attempted to increase power by combining many SNPs into a single score, yet still failed to reveal any significant associations. We considered that there may still be a genetic overlap among the as yet unidentified RA alleles, but found no evidence for this because we did not observe any apparent increment of variance explained in any of the disease activity measures when a genome-wide polygenic risk score model was performed. This might indicate that RA risk alleles do not necessarily have an effect on TNFi treatment, despite most of the current treatment target genes/pathways being involved in general inflammatory response; it may be reasonable to expect more and different biological pathways via which TNFi exerts its effect. In light of the limited statistical power in the current study, however, RA risk SNPs with modest effects in TNFi treatment response cannot be ruled out. One nominally significant finding may deserve mention: rs629326 (located 23.61 kb 5′ of *TAGAP*, involved in T-cell activation) was fairly strongly associated with EULAR response in our material, with OR = 0.31 (0.15–0.62), and FDR-adjusted (for 76 tests) *p* = 0.08. Further, the *HLA-DRB1* SE alleles were associated with reduced EULAR response when restricting the time window to 2–5 months (OR = 0.69 (0.49–0.97), unadjusted *p* = 0.0347), although this was a post-hoc analysis and the *p* value would not remain significant after adjusting for multiple testing. Interestingly, specific *HLA-DRB1* amino acids have been associated previously with TNFi treatment response, where valine at amino acid position 11 was reported to be associated with a smaller change in Larsen score, and improved EULAR response [24]. We unfortunately lacked data on joint erosions. Our results for treatment response, however, did not immediately support the finding that the valine-containing VKA haplotype was associated with a good EULAR response (OR in [24] = 1.23 (1.06–1.43)), while the OR was 1.06 (0.58–1.94) for our data among ACPA-positive RA patients. The confidence intervals overlap greatly, and we are thus unable to either refute or confirm this previous finding. We did find a nominally significant association of residue serine (OR = 1.96 (1.14–3.37)) at position 13 and tightly linked with valine at position 11. This should be interpreted with caution, however, because it was not statistically significant after correcting for the multiplicity of tests. When the haplotype analyses were restricted to patients with high disease activity RA (baseline DAS > 5.1), to maximize comparability with the UK study, the association with valine further diminished (Additional file 11: Table S10).

When addressing continuous measures of treatment response, interestingly, all of the risk scores seemed to consistently explain a small yet significant proportion (5%) of the variance in HAQ changes in ACPA-positive RA. The clinical value of such results warrants further confirmation. It has been suggested that the modest influence of genetic effects on treatment response is due to the “composite” traits of the DAS28 score that most outcomes are based upon, and which relies on information from both subjective and objective measures [26]; and that using a well-defined phenotype could aid in disclosing the true genetic effects [26]. We examined each component of the DAS28 without seeing any remarkable associations, except for a small proportion of variance in ESR change explained by amino acid risk score in ACPA-negative RA, which suffered markedly from limited power.

One strength of the current study is the accuracy of both exposures and outcomes. The genetic data were genotyped and quality controlled, and the amino acid data, although imputed, presented a high concordance rate as compared with true genotyping data (a 97.3% concordance rate for two-digit SE and 95.0% for four-digit SE) [30]. We used clinically relevant outcome measures recommended by the European guidelines collected as part of clinical practice in an unselected manner. Because no environmental confounders could in practice influence the genetic markers, we performed the analyses without including adjustment for many covariates to preserve power. One limitation is insufficient power, which could cause false positive findings, and we consider the overall lack of association to be the main conclusion of this study. Another weakness of this study is the time point at which clinical response to TNFi therapy is determined. In a highly heterogeneous disease like RA, time of clinical assessment is crucial and can substantially modify response classification. However, our register-based data could not provide evaluation at time points as exact as those usually obtained in controlled trials, but rather in a time window, reflecting the Swedish clinical practice. We performed a post-hoc sensitive analysis restricting the time window for the evaluation visit to 2–5 months, and the results remained the same.

## Conclusion

Our negative results imply that there is no strong evidence supporting a significant role of RA risk genes in the response to TNFi treatment; and that none of the SNPs, amino acids, or haplotypes examined in our study seem to be meaningful individual predictors of TNFi therapy, although weak associations cannot be ruled out. Although this suggest that future studies of TNFi response may want to focus on other genetic risk factors, combining genetic information connected to RA onset with clinical, nongenetic predictors, or perhaps their interaction, may also potentially prove valuable.

## Additional files

Additional file 1: Table S1. Presenting characteristics of the 76 SNPs that are known RA risk SNPs. (DOCX 27 kb)
Additional file 2: Table S2. Presenting definitions of amino acid residues and haplotypes. (DOCX 18 kb)
Additional file 3: Table S3. Presenting the distribution of risk scores calculated based on 76 SNPs, each of the four amino acid positions, and the haplotypes. (DOCX 20 kb)
Additional file 4: Table S4. Presenting associations between amino acid GRS and RA risk. (DOCX 18 kb)
Additional file 5: Table S5. Presenting GRS predicting EULAR response, excluding etanercept. (DOCX 17 kb)
Additional file 6: Table S6. Presenting GRS predicting EULAR response, at 6–8 months. (DOCX 17 kb)
Additional file 7: Table S7. Presenting associations between amino acid GRS and EULAR response. (DOCX 18 kb)
Additional file 8: Table S8. Presenting associations between each of the 76 SNPs and EULAR response. (DOCX 37 kb)
Additional file 9: Figure S1. Showing a QQ plot mapping 76 SNPs predicting EULAR response. (DOCX 47 kb)
Additional file 10: Table S9. Presenting associations between individual amino acid residues and EULAR response. (DOCX 23 kb)
Additional file 11: Table S10. Presenting associations between amino acid haplotypes and EULAR response, among patients with DAS28 > 5.1. (DOCX 18 kb)
Additional file 12: Table S11. Presenting associations between amino acid haplotypes and EULAR response, comparing patients with good response with patients with no response. (DOCX 19 kb)
Additional file 13: Table S12. Presenting variance in disease activity changes by risk scores. (DOCX 24 kb)
Additional file 14: Table S13. Presenting associations between GRS SNPs and baseline clinical characteristics. (DOCX 18 kb)
