# Supplementary material for: A genetic risk score composed of rheumatoid arthritis risk alleles, HLA-DRB1 haplotypes, and response to TNFi therapy – results from a Swedish cohort study
Source: Arthritis Res Ther. 2016 Dec 3;18:288. doi: 10.1186/s13075-016-1174-z (PMC5135751; doi:10.1186/s13075-016-1174-z)
Supplement: Additional file 1: Table S1. — Presenting characteristics of the 76 SNPs that are known RA risk SNPs. (DOCX 27 kb) [file 13075_2016_1174_MOESM1_ESM.docx]

A genetic risk score composed of rheumatoid arthritis risk alleles, HLA-DRB1 haplotypes, and response to TNFi therapy – Results from a Swedish cohort study

Xia Jiang^1^, Johan Askling^1,2^, Saedis Saevarsdottir^2^, Leonid Padyukov^2^, Lars Alfredsson^3^, Sebastien Viatte^4^, Thomas Frisell^1^.

1. Unit of Clinical Epidemiology (KEP), Department of Medicine, Karolinska University Hospital.
2. Rheumatology Unit, Department of Medicine Solna, Karolinska Institutet, and Karolinska University Hospital, Stockholm, Sweden.
3. Cardiovascular Unit, Institute of Environmental Medicine, Karolinska Institutet, Stockholm, Sweden.
4. Arthritis Research UK Centre for Genetics and Genomics, Centre for Musculoskeletal Research, Faculty of Biology, Medicine and Health, Manchester Academic Health Science Centre, The University of Manchester, Manchester, Oxford Road, Manchester, M13 9PT, UK

This online supplement contains:

Table S1, Characteristics of the 76 SNPs

| Table S1. Characteristics of the 76 SNPs. | | | | | | | |
| --- | --- | --- | --- | --- | --- | --- | --- |
| Chromosome | SNP* | GRCh37Position | Gene Symbol | Gene Location | RA Locus | Target SNP | r^2^ |
| 1 | rs2843401 | 2528133 | MMEL1 | INTRON |  |  |  |
| 1 | rs2240336 | 17674402 | PADI4 | INTRON | PADI4 |  |  |
| 1 | rs2306627 | 38260503 | MANEAL | INTRON |  | rs24811352 | 0.92 |
| 1 | rs883220 | 38616871 | POU3F1 \| LOC400750 | INTERGENIC |  |  |  |
| 1 | rs2476601 | 114377568 | PTPN22 | COMPLEX | PTPN22 |  |  |
| 1 | rs798000 | 117280696 | IGSF3 \| CD2 | INTERGENIC | CD58 |  |  |
| 1 | rs8192284 | 154426970 | IL6R | CODING |  |  |  |
| 1 | rs3761959 | 157669278 | FCRL3 | INTRON |  | rs2317230 | 0.97 |
| 1 | rs10494360 | 161475750 | FCGR2A | INTRON |  |  |  |
| 1 | rs1557121 | 173353881 | LOC646870 | INTRON |  | rs2105325 | 1 |
| 1 | rs2014863 | 198809601 | PTPRC \| LOC100131234 | INTERGENIC |  |  |  |
| 2 | rs1355208 | 30445322 | YPEL5 \| LBH | INTERGENIC |  | rs10175798 | 1 |
| 2 | rs34695944 | 61124850 | REL | INTRON | REL |  |  |
| 2 | rs6546146 | 65556324 | SPRED2 | INTRON | SPRED2 |  |  |
| 2 | rs10209110 | 100672692 | AFF3 | INTRON | AFF3 |  |  |
| 2 | rs13426947 | 191933254 | STAT4 | INTRON | STAT4 |  |  |
| 2 | rs16837131 | 202173812 | ALS2CR12 | INTRON |  | rs6715284 | 1 |
| 2 | rs1980422 | 204610396 | CD28 \| CTLA4 | INTERGENIC | CD28 |  |  |
| 2 | rs11571302 | 204742934 | CTLA4 \| ICOS | INTERGENIC | CTLA5 |  |  |
| 3 | rs7639882 | 16985272 | PLCL2 | INTRON |  | rs4452313 | 0.92 |
| 3 | rs3806624 | 27764623 | EOMES \| C3orf68 | INTERGENIC |  |  |  |
| 3 | rs35677470 | 58183636 | DNASE1L3 | CODING |  |  |  |
| 4 | rs932036 | 26090862 | LOC389203 \| RBPJ | INTERGENIC | RBPJ |  |  |
| 4 | rs2352593 | 48197767 | TEC | INTRON |  | rs2664035 | 0.62 |
| 4 | rs78560100 | 123041471 | TRPC3 \| KIAA1109 | INTERGENIC | IL2-IL21 |  |  |
| 5 | rs71624119 | 55440730 | ANKRD55 | INTRON | ANKRD55 |  |  |
| 5 | rs39984 | 102597292 | C5orf30 | INTRON | GIN1 |  |  |
| 5 | rs10065782 | 131552658 | P4HA2 | INTRON |  | rs657075 | 0.88 |
| 6 | rs660895 | 32577380 | HLA-DRB1 \| HLA-DQA1 | INTERGENIC |  |  |  |
| 6 | rs7764323 | 36345840 | ETV7 | INTRON |  | rs2234067 | 0.61 |
| 6 | rs6911690 | 106470963 | PREP \| PRDM1 | INTERGENIC |  |  |  |
| 6 | rs9386514 | 106636902 | ATG5 | INTRON |  | rs9372120 | 0.76 |
| 6 | rs6920220 | 138006504 | OLIG3 \| LOC100130476 | INTERGENIC | TNFAIP3 |  |  |
| 6 | rs629326 | 159496713 | LOC727911 \| FNDC1 | INTERGENIC | TAGAP |  |  |
| 6 | rs34046992 | 167537754 | CCR6 | INTRON | CCR7 |  |  |
| 7 | rs67250450 | 28174986 | JAZF1 \| LOC100128081 | INTERGENIC |  |  |  |
| 7 | rs4272 | 92236829 | CDK6 | UTR |  |  |  |
| 7 | rs3807306 | 128580680 | IRF5 | INTRON | IRF5 |  |  |
| 8 | rs4840565 | 11345545 | C8orf13 \| BLK | INTERGENIC | BLK |  |  |
| 8 | rs998731 | 81095395 | LOC100133047 | INTRON |  |  |  |
| 8 | rs678347 | 102463602 | NACAP1 \| GRHL2 | INTERGENIC |  |  |  |
| 8 | rs6651252 | 129567181 | PVT1 \| LOC728724 | INTERGENIC |  | rs1516971 | 0.80 |
| 9 | rs2812378 | 34710260 | CCL21 \| LOC259308 | INTERGENIC | CCL21 |  |  |
| 9 | rs10739580 | 123695282 | TRAF1 \| C5 | INTERGENIC | TRAF1/C5 |  |  |
| 10 | rs10795791 | 6108340 | IL2RA \| RBM17 | INTERGENIC | IL2RA |  |  |
| 10 | rs947474 | 6390450 | LOC399715 \| DKFZp667F0711 | INTERGENIC |  |  |  |
| 10 | rs2275806 | 8095340 | FLJ45983 | UTR |  |  |  |
| 10 | rs793108 | 31415106 | ZNF438 \| LOC100129789 | INTERGENIC |  |  |  |
| 10 | rs2671692 | 50097819 | WDFY4 | INTRON |  |  |  |
| 10 | rs12764378 | 63800004 | ARID5B | INTRON |  |  |  |
| 11 | rs570676 | 36492191 | FLJ14213 \| TRAF6 | INTERGENIC |  |  |  |
| 11 | rs595158 | 60909581 | VPS37C | INTRON |  |  |  |
| 11 | rs968567 | 61595564 | LOC100131326 \| FADS2 | INTERGENIC |  |  |  |
| 11 | rs4409785 | 95311422 | LOC100129203 \| FAM76B | INTERGENIC |  |  |  |
| 11 | rs4938573 | 118741842 | DDX6 \| BLR1 | INTERGENIC |  |  |  |
| 12 | rs10683701 | 58092088 | OS9 | INTRON | KIF5A-PIP4K2C |  |  |
| 12 | rs4766578 | 111904371 | ATXN2 | INTRON |  | rs10774624 | 0.81 |
| 13 | rs7993214 | 40350912 | COG6 \| FOXO1 | INTERGENIC |  | r9603616 | 1 |
| 14 | rs911263 | 68753593 | RAD51L1 | INTRON |  | rs1950897 | 0.96 |
| 15 | rs8043085 | 38828140 | RASGRP1 | INTRON |  |  |  |
| 15 | rs8026898 | 69991417 | LOC145837 \| TLE3 | INTERGENIC |  |  |  |
| 16 | rs17606153 | 11794895 | TXNDC11 | INTRON |  | rs4780401 | 0.70 |
| 16 | rs13330176 | 86019087 | IRF8 \| LOC100131952 | INTERGENIC |  |  |  |
| 17 | rs1877030 | 37740161 | CRKRS \| NEUROD2 | INTERGENIC |  |  |  |
| 17 | rs12936409 | 38043649 | ZPBP2 \| GSDML | INTERGENIC |  |  |  |
| 18 | rs34794968 | 67531026 | CD226 | UTR |  | rs2469434 | 0.93 |
| 19 | rs34536443 | 10463118 | TYK2 | CODING |  |  |  |
| 20 | rs6032662 | 44734310 | NCOA5 \| CD40 | INTERGENIC | CD40 |  |  |
| 21 | rs2834512 | 35911599 | RCAN1 | INTRON |  |  |  |
| 21 | rs9979383 | 36715761 | RUNX1 \| SETD4 | INTERGENIC |  |  |  |
| 21 | rs1893592 | 43855067 | UBASH3A | INTRON |  |  |  |
| 21 | rs2236668 | 45650009 | ICOSLG | INTRON |  |  |  |
| 22 | rs11089637 | 21979096 | UBE2L3 \| LOC150223 | INTERGENIC |  |  |  |
| 22 | rs3218251 | 37545505 | IL2RB | INTRON | IL2RB |  |  |
| 22 | rs909685 | 39747671 | SYNGR1 | INTRON |  |  |  |
| X | rs13397 | 153248248 | TMEM187 | CODING |  |  |  |
| *: For those 16 SNPs on which only proxy SNPs were found and used, their original SNPs were listed in the second last column, with r^2^ showed in the last column. | | | | | | | |
