# Supplementary material for: A genetic risk score composed of rheumatoid arthritis risk alleles, HLA-DRB1 haplotypes, and response to TNFi therapy – results from a Swedish cohort study
Source: Arthritis Res Ther. 2016 Dec 3;18:288. doi: 10.1186/s13075-016-1174-z (PMC5135751; doi:10.1186/s13075-016-1174-z)
Supplement: Additional file 6: Table S6. — Presenting GRS predicting EULAR response, at 6–8 months. (DOCX 17 kb) [file 13075_2016_1174_MOESM6_ESM.docx]

A genetic risk score composed of rheumatoid arthritis risk alleles, HLA-DRB1 haplotypes, and response to TNFi therapy – Results from a Swedish cohort study

Xia Jiang^1^, Johan Askling^1,2^, Saedis Saevarsdottir^2^, Leonid Padyukov^2^, Lars Alfredsson^3^, Sebastien Viatte^4^, Thomas Frisell^1^.

1. Unit of Clinical Epidemiology (KEP), Department of Medicine, Karolinska University Hospital.
2. Rheumatology Unit, Department of Medicine Solna, Karolinska Institutet, and Karolinska University Hospital, Stockholm, Sweden.
3. Cardiovascular Unit, Institute of Environmental Medicine, Karolinska Institutet, Stockholm, Sweden.
4. Arthritis Research UK Centre for Genetics and Genomics, Centre for Musculoskeletal Research, Faculty of Biology, Medicine and Health, Manchester Academic Health Science Centre, The University of Manchester, Manchester, Oxford Road, Manchester, M13 9PT, UK

This online supplement contains:

Table S6, GRS predicting EULAR response, at 2-5 months

| Table S6. The associations between genetic risk scores and TNFi treatment response (achieving good/moderate EULAR response vs. no) at 2-5 months, in RA overall, in by ACPA-status. | | | |
| --- | --- | --- | --- |
| **GRS** | **Overall RA** | **ACPA-positive RA** | **ACPA-negative RA** |
| **GRS_76SNPs_** | | | |
| linear | 0.91 (0.76-1.09) | 0.95 (0.76-1.18) | 0.87 (0.53-1.41) |
| Q1 | ref. | ref. | ref. |
| Q2 vs Q1 | 0.65 (0.28-1.52) | 0.88 (0.27-2.83) | 0.72 (0.18-2.95) |
| Q3 vs Q1 | 0.83 (0.37-1.87) | 1.19 (0.41-3.47) | 0.71 (0.17-2.98) |
| Q4 vs Q1 | 0.65 (0.31-1.39) | 0.85 (0.31-2.32) | 0.87 (0.21-3.67) |
| **GRS_Haplotype_** | | | |
| linear | 0.84 (0.68-1.04) | 0.85 (0.65-1.10) | 1.18 (0.70-2.01) |
| Q1 | ref. | ref. | ref. |
| Q2 vs Q1 | 1.30 (0.53-3.20) | 0.62 (0.15-2.60) | 2.82 (0.75-10.54) |
| Q3 vs Q1 | 1.30 (0.60-2.78) | 0.95 (0.28-3.23) | 3.11 (0.72-13.48) |
| Q4 vs Q1 | 0.91 (0.42-1.97) | 0.65 (0.19-2.25) | 2.99 (0.71-12.59) |
| **Any SE** | 0.69 (0.49-0.97) | 0.69 (0.46-1.05) | 1.04 (0.49-2.22) |
| RA: rheumatoid arthritis; Q: quartile. Odds ratio together with 95% confidence intervals were presented. | | | |
