# Supplementary material for: A genetic risk score composed of rheumatoid arthritis risk alleles, HLA-DRB1 haplotypes, and response to TNFi therapy – results from a Swedish cohort study
Source: Arthritis Res Ther. 2016 Dec 3;18:288. doi: 10.1186/s13075-016-1174-z (PMC5135751; doi:10.1186/s13075-016-1174-z)
Supplement: Additional file 8: Table S8. — Presenting associations between each of the 76 SNPs and EULAR response. (DOCX 37 kb) [file 13075_2016_1174_MOESM8_ESM.docx]

A genetic risk score composed of rheumatoid arthritis risk alleles, HLA-DRB1 haplotypes, and response to TNFi therapy – Results from a Swedish cohort study

Xia Jiang^1^, Johan Askling^1,2^, Saedis Saevarsdottir^2^, Leonid Padyukov^2^, Lars Alfredsson^3^, Sebastien Viatte^4^, Thomas Frisell^1^.

1. Unit of Clinical Epidemiology (KEP), Department of Medicine, Karolinska University Hospital.
2. Rheumatology Unit, Department of Medicine Solna, Karolinska Institutet, and Karolinska University Hospital, Stockholm, Sweden.
3. Cardiovascular Unit, Institute of Environmental Medicine, Karolinska Institutet, Stockholm, Sweden.
4. Arthritis Research UK Centre for Genetics and Genomics, Centre for Musculoskeletal Research, Faculty of Biology, Medicine and Health, Manchester Academic Health Science Centre, The University of Manchester, Manchester, Oxford Road, Manchester, M13 9PT, UK

This online supplement contains:

Table S8, The associations between 76 SNPs and EULAR response

| Table S8. The associations between 76 individual SNPs and EULAR response to TNFi treatment. | | | | | | | | | | |
| --- | --- | --- | --- | --- | --- | --- | --- | --- | --- | --- |
| SNPs | Gene Symbol | Overall RA | p-value | FDR adjusted P* | ACPA-positive RA | p-value | FDR adjusted P* | ACPA-negative RA | p-value | FDR adjusted P* |
| rs2843401 | MMEL1 | 1.19 (0.90-1.58) | 0.22 | 0.72 | 1.25 (0.91-1.73) | 0.17 | 0.77 | 0.89 (0.45-1.77) | 0.75 | 0.94 |
| rs2240336 | PADI4 | 1.12 (0.86-1.46) | 0.42 | 0.83 | 1.14 (0.84-1.56) | 0.39 | 0.86 | 1.12 (0.59-2.12) | 0.73 | 0.94 |
| rs2306627 | MANEAL | 0.82 (0.63-1.06) | 0.13 | 0.72 | 0.78 (0.58-1.06) | 0.11 | 0.77 | 0.84 (0.45-1.57) | 0.58 | 0.92 |
| rs883220 | POU3F1 \| LOC400750 | 0.84 (0.62-1.13) | 0.24 | 0.72 | 0.99 (0.69-1.41) | 0.95 | 0.97 | 0.72 (0.36-1.48) | 0.38 | 0.82 |
| rs2476601 | PTPN22 | 1.25 (0.91-1.71) | 0.17 | 0.72 | 1.15 (0.82-1.62) | 0.42 | 0.86 | 2.11 (0.67-6.68) | 0.20 | 0.77 |
| rs798000 | IGSF3 \| CD2 | 1.27 (0.96-1.67) | 0.09 | 0.72 | 1.18 (0.86-1.62) | 0.29 | 0.86 | 1.73 (0.85-3.53) | 0.13 | 0.76 |
| rs8192284 | IL6R | 0.98 (0.76-1.25) | 0.85 | 0.92 | 1.01 (0.76-1.35) | 0.92 | 0.97 | 0.64 (0.34-1.17) | 0.15 | 0.76 |
| rs3761959 | FCRL3 | 0.88 (0.68-1.13) | 0.31 | 0.72 | 0.76 (0.57-1.01) | 0.06 | 0.76 | 1.59 (0.82-3.07) | 0.17 | 0.76 |
| rs10494360 | FCGR2A | **0.70 (0.49-0.99)** | 0.05 | 0.72 | 0.74 (0.49-1.13) | 0.16 | 0.77 | **0.44 (0.21-0.91)** | 0.03 | 0.59 |
| rs1557121 | LOC646870 | 0.92 (0.68-1.24) | 0.58 | 0.86 | 0.82 (0.57-1.16) | 0.26 | 0.84 | 1.33 (0.66-2.67) | 0.43 | 0.87 |
| rs2014863 | PTPRC \| LOC100131234 | 0.95 (0.73-1.24) | 0.70 | 0.89 | 0.83 (0.62-1.12) | 0.23 | 0.82 | 1.47 (0.77-2.83) | 0.25 | 0.81 |
| rs1355208 | YPEL5 \| LBH | 1.21 (0.94-1.56) | 0.13 | 0.72 | 1.11 (0.83-1.49) | 0.49 | 0.88 | 1.40 (0.77-2.52) | 0.27 | 0.81 |
| rs34695944 | REL | 1.11 (0.80-1.55) | 0.53 | 0.85 | 1.10 (0.75-1.61) | 0.62 | 0.91 | 1.23 (0.52-2.88) | 0.64 | 0.93 |
| rs6546146 | SPRED2 | 0.90 (0.71-1.16) | 0.43 | 0.83 | 0.95 (0.72-1.27) | 0.74 | 0.91 | 0.61 (0.33-1.12) | 0.11 | 0.76 |
| rs10209110 | AFF3 | 0.98 (0.76-1.25) | 0.87 | 0.92 | 0.93 (0.70-1.25) | 0.65 | 0.91 | 1.22 (0.68-2.19) | 0.50 | 0.90 |
| rs13426947 | STAT4 | 0.86 (0.66-1.12) | 0.27 | 0.72 | 0.83 (0.61-1.12) | 0.22 | 0.82 | 1.48 (0.73-3.03) | 0.28 | 0.81 |
| rs16837131 | ALS2CR12 | 1.04 (0.78-1.41) | 0.77 | 0.89 | 1.09 (0.77-1.53) | 0.64 | 0.91 | 0.96 (0.48-1.92) | 0.90 | 0.98 |
| rs1980422 | CD28 \| CTLA4 | 1.03 (0.79-1.33) | 0.85 | 0.92 | 1.09 (0.81-1.47) | 0.58 | 0.91 | 0.75 (0.40-1.40) | 0.37 | 0.82 |
| rs11571302 | CTLA4 \| ICOS | 0.85 (0.66-1.10) | 0.21 | 0.72 | **0.73 (0.54-0.97)** | 0.03 | 0.62 | 1.46 (0.77-2.75) | 0.24 | 0.81 |
| rs7639882 | PLCL2 | 1.16 (0.80-1.68) | 0.44 | 0.83 | 1.28 (0.83-1.99) | 0.27 | 0.84 | 0.86 (0.39-1.93) | 0.72 | 0.94 |
| rs3806624 | EOMES \| C3orf68 | 0.95 (0.68-1.31) | 0.74 | 0.89 | 0.96 (0.66-1.40) | 0.84 | 0.95 | 0.78 (0.36-1.68) | 0.52 | 0.90 |
| rs35677470 | DNASE1L3 | 1.08 (0.78-1.49) | 0.65 | 0.89 | 1.10 (0.75-1.60) | 0.63 | 0.91 | 1.39 (0.66-2.93) | 0.39 | 0.82 |
| rs932036 | LOC389203 \| RBPJ | 0.91 (0.70-1.18) | 0.49 | 0.83 | 1.04 (0.77-1.40) | 0.82 | 0.95 | 0.61 (0.32-1.17) | 0.14 | 0.76 |
| rs2352593 | TEC | 0.82 (0.64-1.05) | 0.11 | 0.72 | 0.82 (0.61-1.09) | 0.17 | 0.77 | 1.08 (0.57-2.04) | 0.82 | 0.97 |
| rs78560100 | TRPC3 \| KIAA1109 | 0.97 (0.74-1.26) | 0.80 | 0.89 | 0.86 (0.63-1.15) | 0.31 | 0.86 | 1.65 (0.85-3.21) | 0.14 | 0.76 |
| rs71624119 | ANKRD55 | 1.21 (0.91-1.61) | 0.20 | 0.72 | 1.26 (0.89-1.79) | 0.19 | 0.78 | 0.86 (0.46-1.61) | 0.64 | 0.93 |
| rs39984 | C5orf30 | 0.79 (0.60-1.05) | 0.11 | 0.72 | 0.80 (0.58-1.10) | 0.17 | 0.77 | 0.97 (0.47-1.98) | 0.93 | 0.98 |
| rs10065782 | P4HA2 | 0.84 (0.65-1.10) | 0.22 | 0.72 | 0.76 (0.56-1.02) | 0.07 | 0.76 | 1.61 (0.79-3.29) | 0.19 | 0.76 |
| rs660895 | HLA-DRB1 \| HLA-DQA1 | 0.93 (0.73-1.19) | 0.57 | 0.86 | 0.90 (0.67-1.20) | 0.47 | 0.88 | 1.10 (0.61-1.96) | 0.76 | 0.94 |
| rs7764323 | ETV7 | **0.70 (0.53-0.93)** | 0.01 | 0.54 | **0.68 (0.50-0.94)** | 0.02 | 0.50 | 0.70 (0.33-1.48) | 0.35 | 0.82 |
| rs6911690 | PREP \| PRDM1 | 1.22 (0.83-1.77) | 0.31 | 0.72 | 1.29 (0.83-2.00) | 0.26 | 0.84 | 1.57 (0.61-4.07) | 0.35 | 0.82 |
| rs9386514 | ATG5 | 1.09 (0.85-1.41) | 0.50 | 0.83 | 1.05 (0.79-1.41) | 0.73 | 0.91 | 1.28 (0.67-2.42) | 0.45 | 0.87 |
| rs6920220 | OLIG3 \| LOC100130476 | 1.15 (0.88-1.49) | 0.30 | 0.72 | 1.07 (0.79-1.45) | 0.64 | 0.91 | 1.68 (0.89-3.17) | 0.11 | 0.76 |
| rs629326 | LOC727911 \| FNDC1 | **0.31 (0.15-0.62)** | 0.00 | 0.08 | **0.27 (0.13-0.59)** | 0.00 | 0.08 | 0.54 (0.09-3.28) | 0.51 | 0.90 |
| rs34046992 | CCR6 | 1.05 (0.81-1.36) | 0.73 | 0.89 | 1.01 (0.74-1.37) | 0.96 | 0.97 | 1.09 (0.60-1.97) | 0.78 | 0.94 |
| rs67250450 | JAZF1 \| LOC100128081 | 1.00 (0.77-1.29) | 0.98 | 0.98 | 0.95 (0.71-1.28) | 0.73 | 0.91 | 1.17 (0.59-2.31) | 0.65 | 0.93 |
| rs4272 | CDK6 | 0.92 (0.71-1.20) | 0.54 | 0.85 | 0.90 (0.66-1.22) | 0.48 | 0.88 | 0.88 (0.46-1.67) | 0.69 | 0.94 |
| rs3807306 | IRF5 | 0.87 (0.68-1.12) | 0.27 | 0.72 | 0.78 (0.58-1.05) | 0.10 | 0.77 | 1.06 (0.59-1.92) | 0.84 | 0.97 |
| rs4840565 | C8orf13 \| BLK | 1.16 (0.86-1.56) | 0.33 | 0.74 | **1.45 (1.01-2.08)** | 0.04 | 0.67 | 0.91 (0.46-1.79) | 0.78 | 0.94 |
| rs998731 | LOC100133047 | 0.88 (0.58-1.34) | 0.55 | 0.85 | 0.92 (0.57-1.48) | 0.73 | 0.91 | 0.40 (0.12-1.29) | 0.13 | 0.76 |
| rs678347 | NACAP1 \| GRHL2 | 0.94 (0.71-1.25) | 0.68 | 0.89 | 0.81 (0.58-1.12) | 0.21 | 0.82 | 1.71 (0.77-3.79) | 0.19 | 0.76 |
| rs6651252 | PVT1 \| LOC728724 | 0.96 (0.75-1.24) | 0.77 | 0.89 | 1.01 (0.75-1.35) | 0.96 | 0.97 | 1.03 (0.55-1.92) | 0.93 | 0.98 |
| rs2812378 | CCL21 \| LOC259308 | 1.16 (0.85-1.58) | 0.36 | 0.78 | 1.07 (0.75-1.52) | 0.72 | 0.91 | 1.72 (0.80-3.72) | 0.16 | 0.76 |
| rs10739580 | TRAF1 \| C5 | 0.78 (0.52-1.15) | 0.21 | 0.72 | 0.81 (0.52-1.28) | 0.37 | 0.86 | 0.48 (0.19-1.22) | 0.12 | 0.76 |
| rs10795791 | IL2RA \| RBM17 | 1.29 (0.98-1.70) | 0.07 | 0.72 | **1.51 (1.09-2.10)** | 0.01 | 0.50 | 1.01 (0.56-1.84) | 0.97 | 0.98 |
| rs947474 | LOC399715 \| DKFZp667F0711 | 0.94 (0.71-1.25) | 0.69 | 0.89 | 0.86 (0.63-1.17) | 0.34 | 0.86 | 1.43 (0.64-3.18) | 0.38 | 0.82 |
| rs2275806 | FLJ45983 | 0.98 (0.74-1.30) | 0.88 | 0.92 | 0.91 (0.65-1.27) | 0.59 | 0.91 | 1.14 (0.60-2.16) | 0.68 | 0.94 |
| rs793108 | ZNF438 \| LOC100129789 | 1.21 (0.88-1.66) | 0,24 | 0.72 | 0.97 (0.68-1.38) | 0,86 | 0.96 | **2.81 (1.17-6.73)** | 0,02 | 0.59 |
| rs2671692 | WDFY4 | 0.79 (0.60-1.04) | 0,10 | 0.72 | 0.80 (0.59-1.09) | 0,15 | 0.77 | 0.86 (0.42-1.78) | 0,69 | 0.94 |
| rs12764378 | ARID5B | 0.90 (0.68-1.17) | 0,42 | 0.83 | 0.92 (0.67-1.25) | 0,59 | 0.91 | 1.03 (0.54-1.99) | 0,92 | 0.98 |
| rs570676 | FLJ14213 \| TRAF6 | 1.10 (0.83-1.46) | 0,49 | 0.83 | 1.03 (0.74-1.42) | 0,88 | 0.97 | 1.25 (0.63-2.50) | 0,52 | 0.90 |
| rs595158 | VPS37C | 1.24 (0.96-1.58) | 0,10 | 0.72 | 1.12 (0.84-1.50) | 0,43 | 0.86 | 1.75 (0.95-3.20) | 0,07 | 0.76 |
| rs968567 | LOC100131326 \| FADS2 | 0.69 (0.44-1.08) | 0,11 | 0.72 | 0.67 (0.40-1.12) | 0,12 | 0.77 | 0.97 (0.29-3.25) | 0,97 | 0.98 |
| rs4409785 | LOC100129203 \| FAM76B | 1.06 (0.81-1.39) | 0,68 | 0.89 | 1.18 (0.87-1.62) | 0,29 | 0.86 | 0.84 (0.44-1.59) | 0,58 | 0.92 |
| rs4938573 | DDX6 \| BLR1 | **1.34 (1.01-1.77)** | 0,04 | 0.72 | 1.24 (0.91-1.70) | 0,17 | 0.77 | 1.51 (0.72-3.15) | 0,28 | 0.81 |
| rs10683701 | OS9 | 0.74 (0.48-1.16) | 0,19 | 0.72 | 0.68 (0.40-1.14) | 0,14 | 0.77 | 1.00 (0.29-3.43) | 1,00 | 1.00 |
| rs4766578 | ATXN2 | 0.95 (0.69-1.31) | 0,76 | 0.89 | 0.88 (0.61-1.28) | 0,50 | 0.88 | 1.47 (0.62-3.49) | 0,38 | 0.82 |
| rs7993214 | COG6 \| FOXO1 | 1.06 (0.79-1.41) | 0,70 | 0.89 | 1.04 (0.75-1.45) | 0,81 | 0.95 | 1.40 (0.69-2.84) | 0,34 | 0.82 |
| rs911263 | RAD51L1 | 0.85 (0.56-1.29) | 0,45 | 0.83 | 1.00 (0.62-1.62) | 0,99 | 0.99 | **0.28 (0.10-0.78)** | 0,01 | 0.59 |
| rs8043085 | RASGRP1 | 0.99 (0.74-1.32) | 0,93 | 0.95 | 1.14 (0.82-1.60) | 0,43 | 0.86 | 0.92 (0.44-1.92) | 0,83 | 0.97 |
| rs8026898 | LOC145837 \| TLE3 | 1.40 (0.87-2.26) | 0,17 | 0.72 | 1.32 (0.75-2.34) | 0,34 | 0.86 | 1.38 (0.50-3.79) | 0,53 | 0.90 |
| rs17606153 | TXNDC11 | 0.98 (0.64-1.50) | 0,93 | 0.95 | 1.06 (0.67-1.69) | 0,81 | 0.95 | 0.96 (0.28-3.29) | 0,95 | 0.98 |
| rs13330176 | IRF8 \| LOC100131952 | 0.99 (0.72-1.34) | 0,92 | 0.95 | 1.07 (0.74-1.53) | 0,73 | 0.91 | 0.67 (0.32-1.43) | 0,31 | 0.82 |
| rs1877030 | CRKRS \| NEUROD2 | 0.95 (0.70-1.29) | 0,74 | 0.89 | 1.02 (0.72-1.45) | 0,91 | 0.97 | 0.97 (0.46-2.04) | 0,93 | 0.98 |
| rs12936409 | ZPBP2 \| GSDML | 1.16 (0.89-1.52) | 0,27 | 0.72 | 1.17 (0.86-1.59) | 0,32 | 0.86 | 1.18 (0.62-2.27) | 0,61 | 0.93 |
| rs34794968 | CD226 | 0.96 (0.76-1.23) | 0,76 | 0.89 | 1.06 (0.81-1.41) | 0,66 | 0.91 | 0.71 (0.39-1.28) | 0,25 | 0.81 |
| rs34536443 | TYK2 | 0.85 (0.63-1.15) | 0,30 | 0.72 | 0.88 (0.62-1.25) | 0,47 | 0.88 | 0.82 (0.41-1.64) | 0,58 | 0.92 |
| rs6032662 | NCOA5 \| CD40 | 0.96 (0.71-1.29) | 0,77 | 0.89 | 1.02 (0.73-1.43) | 0,90 | 0.97 | 0.83 (0.40-1.72) | 0,62 | 0.93 |
| rs2834512 | RCAN1 | 1.16 (0.91-1.48) | 0,24 | 0.72 | 1.10 (0.83-1.45) | 0,51 | 0.88 | **2.08 (1.07-4.05)** | 0,03 | 0.59 |
| rs9979383 | RUNX1 \| SETD4 | 0.83 (0.59-1.15) | 0,26 | 0.72 | 0.90 (0.62-1.32) | 0,59 | 0.91 | 0.59 (0.28-1.24) | 0,16 | 0.76 |
| rs1893592 | UBASH3A | 0.95 (0.74-1.22) | 0,67 | 0.89 | 1.04 (0.78-1.40) | 0,78 | 0.94 | 0.72 (0.39-1.35) | 0,31 | 0.82 |
| rs2236668 | ICOSLG | 0.88 (0.67-1.16) | 0,37 | 0.78 | 0.87 (0.64-1.19) | 0,39 | 0.86 | 0.76 (0.38-1.54) | 0,44 | 0.87 |
| rs11089637 | UBE2L3 \| LOC150223 | 0.95 (0.63-1.42) | 0,80 | 0.89 | 0.88 (0.56-1.39) | 0,60 | 0.91 | 2.47 (0.66-9.20) | 0,18 | 0.76 |
| rs3218251 | IL2RB | 0.87 (0.67-1.12) | 0,28 | 0.72 | 0.95 (0.71-1.28) | 0,74 | 0.91 | 0.79 (0.41-1.49) | 0,46 | 0.87 |
| rs909685 | SYNGR1 | 1.10 (0.84-1.44) | 0,49 | 0.83 | 1.15 (0.84-1.56) | 0,39 | 0.86 | 0.90 (0.49-1.65) | 0,74 | 0.94 |
| rs13397 | TMEM187 | 1.12 (0.82-1.51) | 0,48 | 0.83 | 1.15 (0.81-1.64) | 0,43 | 0.86 | 0.98 (0.49-1.96) | 0,95 | 0.98 |

*) The number of tests was set to 76 for the FDR correction, and made with function p.adjust in R.
