# Supplementary material for: A genetic risk score composed of rheumatoid arthritis risk alleles, HLA-DRB1 haplotypes, and response to TNFi therapy – results from a Swedish cohort study
Source: Arthritis Res Ther. 2016 Dec 3;18:288. doi: 10.1186/s13075-016-1174-z (PMC5135751; doi:10.1186/s13075-016-1174-z)
Supplement: Additional file 13: Table S12. — Presenting variance in disease activity changes by risk scores. (DOCX 24 kb) [file 13075_2016_1174_MOESM13_ESM.docx]

A genetic risk score composed of rheumatoid arthritis risk alleles, HLA-DRB1 haplotypes, and response to TNFi therapy – Results from a Swedish cohort study

Xia Jiang^1^, Johan Askling^1,2^, Saedis Saevarsdottir^2^, Leonid Padyukov^2^, Lars Alfredsson^3^, Sebastien Viatte^4^, Thomas Frisell^1^.

1. Unit of Clinical Epidemiology (KEP), Department of Medicine, Karolinska University Hospital.
2. Rheumatology Unit, Department of Medicine Solna, Karolinska Institutet, and Karolinska University Hospital, Stockholm, Sweden.
3. Cardiovascular Unit, Institute of Environmental Medicine, Karolinska Institutet, Stockholm, Sweden.
4. Arthritis Research UK Centre for Genetics and Genomics, Centre for Musculoskeletal Research, Faculty of Biology, Medicine and Health, Manchester Academic Health Science Centre, The University of Manchester, Manchester, Oxford Road, Manchester, M13 9PT, UK

This online supplement contains:

Table S12, Variance explained in disease activity changes by risk scores

| Table S12. Variance explained in disease activity changes by genetic risk scores on amino acid positions, haplotypes and SE. | | | | | | |
| --- | --- | --- | --- | --- | --- | --- |
| Changes from baseline values | Overall RA | | ACPA-positive RA | | ACPA-negative RA | |
|  | R^2^ | p | R^2^ | p | R^2^ | p |
| **GRS_AAP11_** | | | | | | |
| ΔDAS28 | 0,015037 | 0,16 | 0,015880 | 0,31 | 0,035499 | 0,66 |
| ΔCRP | 0,006324 | 0,74 | 0,005484 | 0,90 | 0,102608 | 0,05 |
| ΔESR | 0,004999 | 0,85 | 0,001895 | 1,00 | **0,106857** | 0,04 |
| ΔSJC | 0,001807 | 0,99 | 0,008731 | 0,72 | 0,040874 | 0,57 |
| ΔTJC | 0,006092 | 0,75 | 0,005282 | 0,91 | 0,020079 | 0,90 |
| ΔVAS Global | 0,010087 | 0,42 | 0,013316 | 0,44 | 0,018067 | 0,93 |
| ΔVAS Pain | 0,013813 | 0,21 | 0,016425 | 0,29 | 0,023014 | 0,87 |
| ΔHAQ | 0,019334 | 0,08 | **0,038986** | 0,01 | 0,020331 | 0,91 |
| **GRS_AAP13_** | | | | | | |
| ΔDAS28 | 0,013258 | 0,23 | 0,014744 | 0,37 | 0,036889 | 0,64 |
| ΔCRP | 0,005810 | 0,78 | 0,005417 | 0,91 | 0,097716 | 0,06 |
| ΔESR | 0,005436 | 0,82 | 0,001912 | 1,00 | **0,112154** | 0,03 |
| ΔSJC | 0,001633 | 0,99 | 0,008453 | 0,74 | 0,043595 | 0,52 |
| ΔTJC | 0,006551 | 0,71 | 0,005414 | 0,90 | 0,018444 | 0,92 |
| ΔVAS Global | 0,008381 | 0,55 | 0,012164 | 0,50 | 0,019264 | 0,91 |
| ΔVAS Pain | 0,012600 | 0,27 | 0,015692 | 0,33 | 0,020564 | 0,90 |
| ΔHAQ | 0,019929 | 0,07 | **0,040052** | 0,01 | 0,022153 | 0,89 |
| **GRS_AAP71_** | | | | | | |
| ΔDAS28 | 0,012448 | 0,27 | 0,010343 | 0,62 | 0,056738 | 0,33 |
| ΔCRP | 0,004209 | 0,90 | 0,004609 | 0,94 | 0,086089 | 0,11 |
| ΔESR | 0,006426 | 0,75 | 0,006655 | 0,86 | 0,042142 | 0,57 |
| ΔSJC | 0,003532 | 0,93 | 0,006882 | 0,83 | 0,047421 | 0,46 |
| ΔTJC | 0,005568 | 0,79 | 0,005019 | 0,92 | 0,045949 | 0,49 |
| ΔVAS Global | 0,006278 | 0,73 | 0,008121 | 0,76 | 0,014973 | 0,96 |
| ΔVAS Pain | 0,011880 | 0,31 | 0,014898 | 0,36 | 0,035577 | 0,66 |
| ΔHAQ | **0,021816** | 0,05 | **0,048649** | 0,00 | 0,038558 | 0,64 |
| **GRS_AAP74_** | | | | | | |
| ΔDAS28 | 0,012764 | 0,25 | 0,022290 | 0,11 | 0,025064 | 0,84 |
| ΔCRP | 0,005014 | 0,84 | 0,005204 | 0,92 | 0,080421 | 0,15 |
| ΔESR | 0,004517 | 0,89 | 0,004257 | 0,96 | 0,053381 | 0,40 |
| ΔSJC | 0,000932 | 1,00 | 0,011916 | 0,52 | 0,037975 | 0,62 |
| ΔTJC | 0,004496 | 0,87 | 0,006792 | 0,83 | 0,024196 | 0,85 |
| ΔVAS Global | 0,007040 | 0,67 | 0,013255 | 0,44 | 0,037990 | 0,62 |
| ΔVAS Pain | 0,013782 | 0,21 | 0,019735 | 0,18 | 0,024265 | 0,85 |
| ΔHAQ | **0,022176** | 0,04 | **0,039197** | 0,01 | 0,013787 | 0,97 |
| **GRS_Haplotype_** | | | | | | |
| ΔDAS28 | 0,014598 | 0,17 | 0,015135 | 0,35 | 0,043732 | 0,52 |
| ΔCRP | 0,005973 | 0,77 | 0,004018 | 0,96 | 0,097816 | 0,06 |
| ΔESR | 0,005958 | 0,79 | 0,002205 | 0,99 | 0,099085 | 0,05 |
| ΔSJC | 0,001993 | 0,99 | 0,007957 | 0,77 | 0,040869 | 0,57 |
| ΔTJC | 0,005449 | 0,80 | 0,004997 | 0,92 | 0,019837 | 0,91 |
| ΔVAS Global | 0,011267 | 0,34 | 0,014777 | 0,36 | 0,025903 | 0,82 |
| ΔVAS Pain | 0,014911 | 0,17 | 0,016788 | 0,28 | 0,033814 | 0,69 |
| ΔHAQ | 0,019944 | 0,07 | **0,040434** | 0,01 | 0,024093 | 0,87 |
| **SE** | | | | | | |
| ΔDAS28 | 0,014126 | 0,17 | 0,015253 | 0,29 | 0,018547 | 0,89 |
| ΔCRP | 0,003071 | 0,93 | 0,002951 | 0,97 | 0,073805 | 0,17 |
| ΔESR | 0,005442 | 0,76 | 0,002444 | 0,98 | 0,066056 | 0,22 |
| ΔSJC | 0,000980 | 1,00 | 0,006043 | 0,82 | 0,040081 | 0,53 |
| ΔTJC | 0,008375 | 0,49 | 0,009328 | 0,61 | 0,017227 | 0,90 |
| ΔVAS Global | 0,008808 | 0,46 | 0,010394 | 0,55 | 0,014008 | 0,94 |
| ΔVAS Pain | 0,012017 | 0,26 | 0,015189 | 0,30 | 0,014837 | 0,93 |
| ΔHAQ | 0,018022 | 0,09 | **0,037972** | 0,01 | 0,010597 | 0,97 |
